# Supplementary material for: Impacts of a DUF2207 Family Protein on Streptococcus mutans Stress Tolerance Responses and Biofilm Formation
Source: Microorganisms. 2023 Aug 1;11(8):1982. doi: 10.3390/microorganisms11081982 (PMC10457818; doi:10.3390/microorganisms11081982)
Supplement: Supplementary file 1 [file microorganisms-11-01982-s001.zip › microorganisms-2521699-supplementary.pdf]

Impacts of a DUF2207 Family Protein on *Streptococcus mutans* Stress Tolerance Responses and Biofilm Formation

Xiaochang Huang[1], Camile G. Laird[1], Paul P. Riley[1], Zezhang Tom Wen[1,2*]

[1]Department of Oral and Craniofacial Biology, School of Dentistry, Louisiana State University Health Sciences Center, New Orleans, LA, USA, and [2] Department of Microbiology, Immunology and Parasitology, School of Medicine, Louisiana State University Health Sciences Center, New Orleans, LA USA, 70112

*Corresponding author: zwen@lsuhsc.edu; phone: (504)9418465; Fax: (504)9418282.

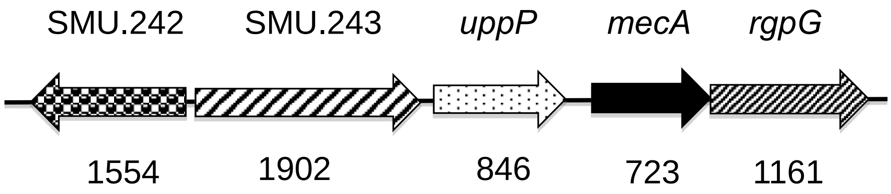

Figure S1. The schematic diagram of the SMU.243 locus and its flanking region in *S. mutans* UA159. Arrows indicate the orientation of the different genes. The locus number and the name of the gene are presented above the arrows, while the numbers underneath represent the size in basepair of the respective genes.

Table S1. Primers used in this study

Names	Forward (5' to 3')	Reverse (5' to 3')	Application
243Fw	ttcggagacataagcatcaataac	atcactcgagatagttgtgacatgg	5' fragment of Δ*SMU.243::nps*
243Rv	atttctagagagaatatgattcgtgac	aatgaaaagttgactaccagttag	3' fragment of Δ*SMU.243::nps*
uppPFw	attgatcaaggaaaactagtcattg	actaaaatctctagaccagtacttgag	5' fragment for Δ*uppP::nps*
uppPRv	actttctcgaggaaataccgtattg	actaaaggagtcaaaacaagtg	3' fragment for Δ*uppP*::*nps*
243Cp1	tagagctcataagcatcaataacattagaag	actaaaatcaggtacccagtacttgag	smu.243 complementation via pBGK3
243Cp2	agacataagcttcaataacattagaag	actaaaatcaagagctcagtacttgag	smu.243 complementation via pDL278
243Cd	gggcagcaatgagacagaaa	tccgtaaagggtggcataga	ΔSMU.243 confirmation
243Cf	accgacaagtcaagcaagag	actaaaatcaaatgcccagtacttgag	ΔSMU.243 confirmation
UppPCf	gagccttctaacttagtaaacaag	ttcttctaaatcttccatactgatggtg	Δ*uppP* confirmation
UppPCp	ttggagctcgtatggagaagttaga	ttcttctaaaggtaccatactgatggtg	Δ*uppP* complementation
UppPCd	cagctgtgattggcctcata	caaggctagcaccaaacataatc	Δ*uppP* confirmation
NPS	ttcttgtttgggaggatgattccac	atattgcgggaaatgcagtggctg	Sequence confirmation
RT	gctaatcattgcaagcagtggtatcaacgcagagtacatggg	aagtctgatattataatcaacatcc	Reverse transcription in RACE
TR-PCR	cattgcaagcagtggtatcaac	agctgaaacaaagctagttgttac	PCR amplification in RACE

Note: Sequences underlined are restriction sites engineered for cloning.

Figure S2: Corrected sequence of the SMU.243 and its promoter region.

Figure S2A. Complete coding sequence of SMU.243 (GeneBank #MW715639.1):

AGGATGTTGATTATAATATCAGACTTTATTGGGGAAGGCTGACACTTAATAAAGATAAT
ACAGCGACTTTTCAACAGGATCTTGTTTATGATTTTGCTTCATCCTACAATGGCCAATA
CGTTACCTTGGGTTCAGCAGGCAATGTACCTAAAGGATTTAAAATTAATAGCAATCCTG
AAGTGAAAGCCTATGAGGTTAACGAGCAGGGAAAATTGACAAAACGCCCGATAAAGACC
AAAATAGAACAGCTGTCAGATGGCTATCGCGCTAAAGTCTATAATGGCGGACATAGCGG
TGATCGTGTTGTCCTTTCTTTAAAGTGGAAACTTCACCATGTCACAACTATTTATTCTG
ATATTGCTGAACTTAATTGGACACCGATTAGTGATTGGGATGCTCCTTTGGATAAGGTC
GTCCTGACAGTTAAGGGACCTTCAAATTCGCTGGCTGTAAGCAGGTTCCATGCTCATAC
AGGCTACTTTAAAAAGCAGCCTAAAGTCACTCAAAAAAATGGAAACTATGAAGTTAATA
TTGAGGGTCTTGGCAAGAATAAAAAGTTGGAACTGCATGCTTATTGGAAACGTTCGGAT
TTTGCTGCTGCCACCGACAAGTCAAGCAAGAGATTATCAAAGTTTCAAGCTCTTGAAGC
TAAAATTGCCAGACGTCAGAAATTTTATCCTCTTTTAGTCGGCTGGCTGTTTCCGATGG
TCAGCGCCATCCTTATCCTTATTTCTATTGTTCTCTATGCTACTTGGAAAGTGGTATTA
GGACAGAGAAAAACAGCAAGGCATATGCATTTATTCAGTCCGCCGGCTGACTTATCCCC
TTTGATTCTTTCTCGTTATGTTTATGATTTAGAAATTCAAGAGTTATCTCCTTTGAAAA
CTAAGCGGAAGCGATATGATTTAGGTTTTAAACAGCTGATTCAAGCTAGCCTCTTAGAT
TTGATTGATCAAGGAAAACTAGTCATTGCAGATGATCATAAATCTTTTTTTGTTCCTGA
TTGGAATAGACTAGAAAGCTATGAAAAGAGATTTCTTATTTTTGTTTATGGCGATAATA
AAAAGACCATGCCTATTGATGGTGCTTTTGATGATTATAAAATTGACAAAAGCATTTTC
AAGGGCAGCAATGAGACAGAAATTCGTAGACGGGGCGGTGATATTCTCCATTTGTTTGA
AAATCGTATGGAGAAGTTAGATAAGGCTGTTAAAAATAAAATAAGCTCTTTGAGTTTGA
ATGATATTCATCGTGAGAGAACGGGCGAAGAAAAGACTCAGCTTATCTTGGTTTATTTC
TTTGCATCTACTGCCATGTTGTTTGCCTTTTTTGTAGGAGCTTTTGCTCTTATCAAGGG
TTATTGGCTGGGGCTTGGGACCAATCTGTTATTGCTTATTGTAGCCAGTCTTTTTCTGA
TTTTCTATCGTCGAAAAGATGATTATTATAAGGTTTCAAGTCTTCTTACGCAAGAAGGC
CTTGCTATCAAACAGGGTTGGGATTCTTTTGAGAATATGATTCGTGACATTAAGAGATT
TGATGATGTCGAACTTGAAGGGGTTATTATCTGGAACCGTATTTTGGTCTATGCCACCC
TTTACGGATATGCTGAACGTGTTCAAAATTATTTGAAAGTTAAAAATATTCATTTGCAG
AACCCACAAATGAATACTTATCTTGAGATCAACCCGAGTTACTATGTGGGACAGTCAAC
CGCAGATTTATCAACCTATACCTCAACTGCTACGAGTGCTTCAAACTTTTCGGTATCGT
CTGGCGGCTCAAGCGGCGGTGGTTTCTCCGGAGGCGGAGGCGGTGGAGGCGGCGGAGCC
TTCTAA

Figure S2B. The translated amino acid sequence of SMU.243:

MKKLLLFLATCLSLVTTSFVSAKDVDYNIRLYWGRLTLNKDNTATFQQDLVYDFASSYN
GQYVTLGSAGNVPKGFKINSNPEVKAYEVNEQGKLTKRPIKTKIEQLSDGYRAKVYNGG
HSGDRVVLSLKWKLHHVTTIYSDIAELNWTPISDWDAPLDKVVLTVKGPSNSLAVSRFH
AHTGYFKKQPKVTQKNGNYEVNIEGLGKNKKLELHAYWKRSDFAAATDKSSKRLSKFQA
LEAKIARRQKFYPLLVGWLFPMVSAILILISIVLYATWKVVLGQRKTARHMHLFSPPAD
LSPLILSRYVYDLEIQELSPLKTKRKRYDLGFKQLIQASLLDLIDQGKLVIADDHKSFF

VPDWNRLESYEKRFLIFVYGDNKKTMPIDGAFDDYKIDKSIFKGSNETEIRRRGGDILH
LFENRMEKLDKAVKNKISSLSLNDIHRERTGEEKTQLILVYFFASTAMLFAFFVGAFAL
IKGYWLGLGTNLLLLIVASLFLIFYRRKDDYYKVSSLLTQEGLAIKQGWDSFENMIRDI
KRFDDVELEGVIIWNRILVYATLYGYAERVQNYLKVKNIHLQNPQMNTYLEINPSYYVG
QSTADLSTYTSTATSASNFSVSSGGSSGGGFSGGGGGGGGGAF*

Figure S2C. The Promoter region of SMU.243:

ATAAGCATCAATAACATTAGAAGCTAAGGCCTGTCTCATCTGAGAGAAGTCTCCCATAG
CTGTTTGCCGAGATACTTTAGGAATCTGATTAATCAAATTATAAAGATAAACACCTTGT
TGGGAGGTCACCTTTGCGCCTGAGAAGTCATTGAGATTTTTGGCTTTGGCATACTTACT
GTCTTTGCGTACAACAAGAACAGGCTCGCTAGTATAGTAGCTATTAGAAAAGGCAATTT
CTTTTTTGCGTTCTTTGGTTGGACTCATTCCAGCGATAATCAGATCAATTTTTCCGGAT
GTTAGGGCTGGCACAAGTCCTTCCCATTTTGTTTTAACAATGAGTGGTTTTTTGCCCAG
AGTTTTAGCAATTTTTTTGGCTGTTTGAACATCGTATCCGTTTGCGTATTGCTTTGTTC
CTTCAATAGGTACAGCCCCATTGCTGCTATTGTCTTGGGTCCAGTTGAAAGGCGCATAA
GCTGCTTCCATTCCAACACGTAGGTAGTTGTCTGCCTGAGCATTTGTCACACCAATAAA
CAGCATGAAAAGGGCAGCCAGACAGCTTAAAATTGTCTTTTTCATTCAGAAGACTCCTG
TATAAAATTTTTTACTTTATCATTTTACAGCAAAAAGTAGCGTATTTCAATATCACTAT
CTGAAAATAGAAGGTTTATCTTTGACTGGGTTTTATGATAAAATAAAAA**A**AGAATTCTT
-35 extended-10 TIS

GATATACTGAGGTTTATAA(ATG)
RBS

Notes: (ATG) indicates the translational start site; TIS is for the transcription initiation site as determined by promoter mapping; RBS, putative ribosome binding site; extended -10 and -35 are the predicted -10 and -35 site, respectively.

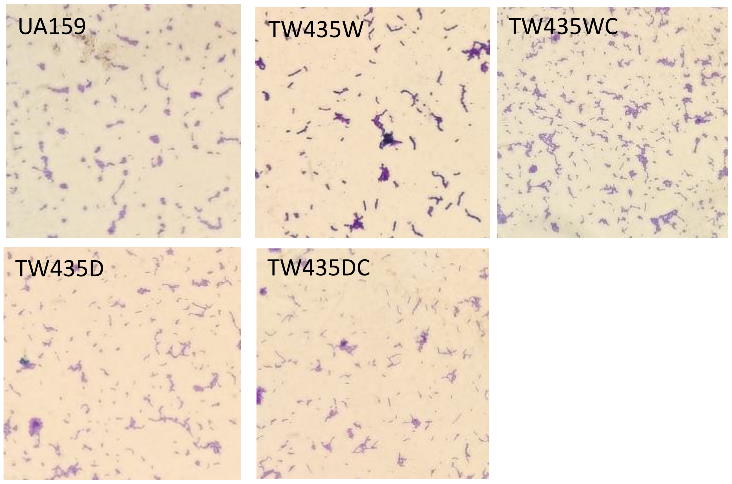

Figure S3. Optical microscopic analysis. *S. mutans* UA159 (UA159), the “rough” (TW435D) and the “smooth” (TW435W) *uppP* mutants, and their respective complement strain, TW435DC and TW435WC were grown in regular BHI broth with proper antibiotic(s). When reaching optical density at 600nm of 0.4, the bacterial cells were stained with 0.1% crystal violet for 30 minutes and washed with phosphate buffered saline, pH 7.0 before analysis using a phase contrast optical microscope. Images were taken with 1000x.

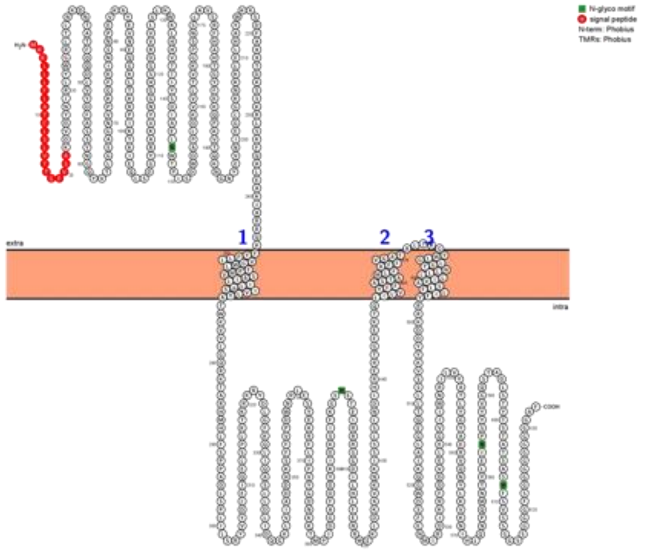

Figure S4. Schematic diagram of the translated protein of SMU.243, when analyzed via Protter, a protein topology analysis tool (wlab.ethz.ch/protter/start).

Structure and function analysis of *S. mutans* SMU.243 and the DUF2207 family of proteins:

Based on NCBI Conserved Domain Database and EMBL-EBI *(http://pfam-legacy.xfam.org/family/PF09972#tabview=tab1)*,
the DUF2207 Superfamily of Proteins are widespread. In bacteria, the genes for proteins with the DUF2207 domain are frequently linked with those for membrane transporters and signal transduction apparatus, indicative of potential roles in material translocation across the membrane and /or signaling and signal transduction. However, the precise function of these proteins in cellular processes await further investigation.

The protein structure of SMU.243 was analyzed using "alphafold2", which is an artificial intelligence system commonly used for protein structure prediction.

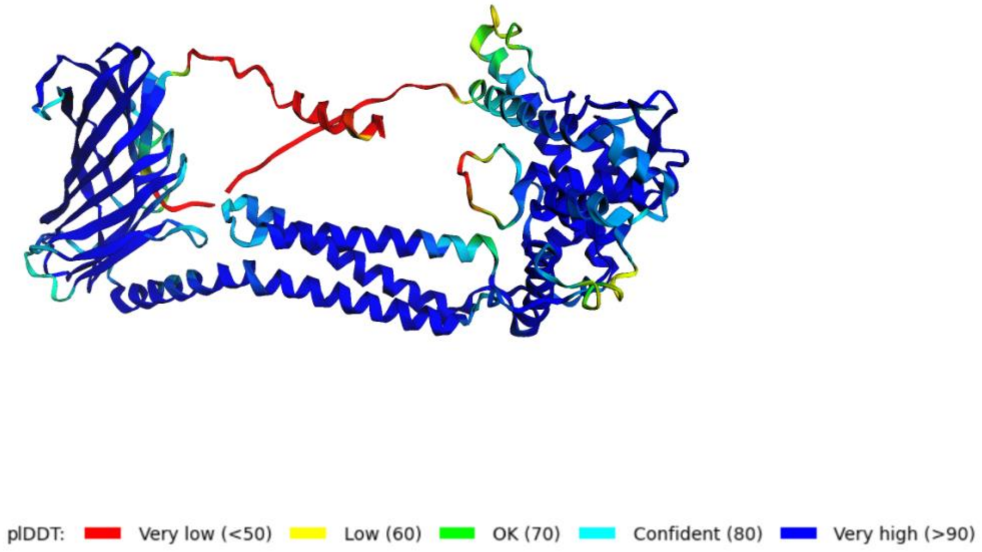

Figure S5. Predicted protein structure via alphafold2.

The predicted protein structure, as shown in Figure S5 above, was then used for Go term functional prediction using DeepFRI. As listed in the tables below, the results indicate that an intrinsic component of membrane, the product of SMU.243 more likely plays a role as transmembrane transporter. Results can be also found by click this link: https://beta.deepfri.flatironinstitute.org/workspace/2SQKNQ/predictions/85A773

Table S2a. Structure-Based Molecular Function - GO Term Predictions

Names	Go Term	score
inorganic molecular entity transmembrane transporter activity	GO:0015318	0.84
transmembrane transporter activity	GO:0022857	0.83
ion transmembrane transporter activity	GO:0015075	0.81
ion binding		
organic cyclic compound binding	GO:0097159	0.53
heterocyclic compound binding	GO:1901363	0.51

Table S2b. Structure-Based Biological Process - GO Term Predictions

name	Go term	score
transmembrane transport	GO:0055085	0.99
ion transport	GO:0006811	0.99
transport	GO:0006810	0.99
establishment of localization	GO:0051234	0.99
ion transmembrane transport	GO:0034220	0.98
cation transport	GO:0006812	0.95
inorganic cation transmembrane transport	GO:0098662	0.92
monovalent inorganic cation transport	GO:0015672	0.87
metal ion transport	GO:0030001	0.58

Table S2c. Structure-Based Cellular Component - GO Term Predictions

Name	Go Term	Score
membrane	GO:0016020	1.00
intrinsic component of membrane	GO:0031224	0.97
integral component of membrane	GO:0016021	0.97
cell periphery	GO:0071944	0.83
plasma membrane	GO:0005886	0.83
membrane	GO:0016020	1.00

Structure-Based Enzyme Commission - GO Term Predictions

No predictions above threshold.

Sequence-Based Molecular Function - GO Term Predictions

No predictions above threshold

Table S2d. Sequence-Based Biological Process - GO Term Predictions

Name	Go Term	Score
organic substance metabolic process	GO:0071704	0.77
cellular metabolic process	GO:0044237	0.73
primary metabolic process	GO:0044238	0.70
nitrogen compound metabolic process	GO:0006807	0.63
organic substance metabolic process	GO:0071704	0.77
cellular metabolic process	GO:0044237	0.73
macromolecule metabolic process	GO:0043170	0.57

Figure S6. Phylogenetic tree of selected DUF2207 family proteins

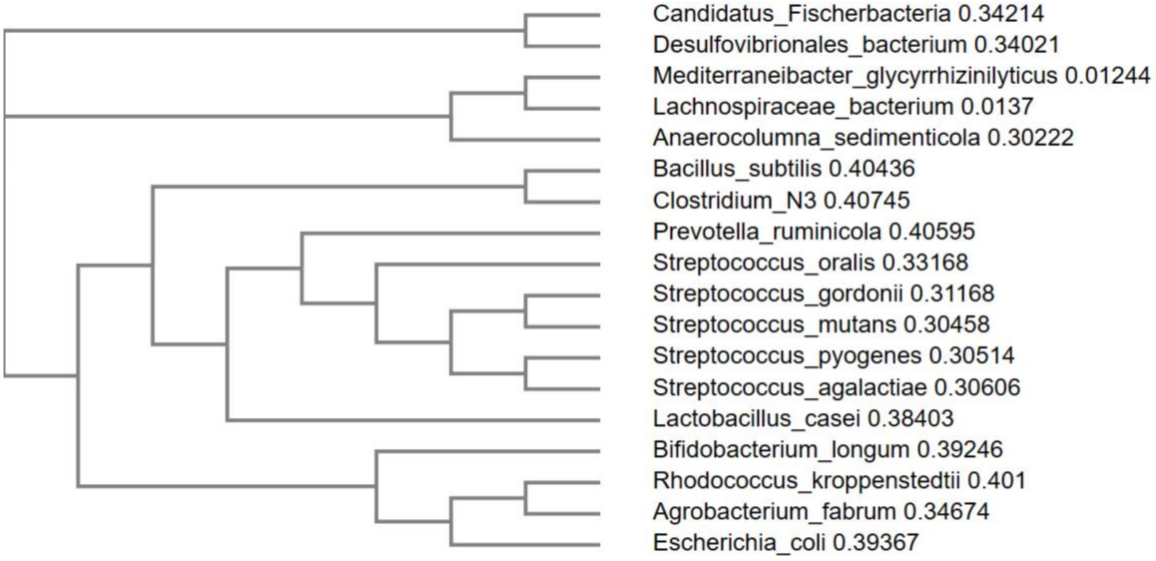

Note: Phylogenetic analysis was carried using Clustal Omega, a multiple Sequence Alignment tool provided by EBI (https://www.ebi.ac.uk/Tools/msa/clustalo/). The amino acid sequences of the proteins analyzed are listed below.

```
>Streptococcus mutans UA159
MKKLLLFLATCLSLVTTSFVSAKDVDYNIRLYWGRLTLNKDNTATFQQDLVYDFASSYNGQYVTLGSAGNV
PKGFKINSNPEVKAYEVNEQGKLTKRPIKTKIEQLSDGYRAKVYNGGHSGDRVVLSLKWKLHHVTTIYSDI
AELNWTPISDWDAPLDKVVLTVKGPSNSLAVSRFHAHTGYFKKQPKVTQKNGNYEVNIEGLGKNKKLELHA
YWKRSDFAAATDKSSKRLSKFQALEAKIARRQKFYPLLVGWLFPMVSAILILISIVLYATWKVVLGQRKTA
RHMHLFSPPADLSPLILSRYVYDLEIQELSPLKTKRKRYDLGFKQLIQASLLDLIDQGKLVIADDHKSFFV
PDWNRLESYEKRFLIFVYGDNKKTMPIDGAFDDYKIDKSIFKGSNETEIRRRGGDILHLFENRMEKLDKAV
KNKISSLSLNDIHRERTGEEKTQLILVYFFASTAMLFAFFVGAFALIKGYWLGLGTNLLLLIVASLFLIFY
RRKDDYYKVSSLLTQEGLAIKQGWDSFENMIRDIKRFDDVELEGVIIWNRILVYATLYGYAERVQNYLKVK
NIHLQNPQMNTYLEINPSYYVGQSTADLSTYTSTATSASNFSVSSGGSSGGGFSGGGGGGGGGAF

>Clostridium N3
MKKVVAVLLFFSIFLSPFIVKADSKSYTIENLVVNSTILRNGDVYVEEELTYYFNGDFNGIYRNLSKKGAD
NITISEVLVEDNKGNVITLREDKGSNNNYYELDNSSSLSKIKIFSKSSNERKTFIFKYTIHGAVVKREDIG
ELYWAFYEVENNIPVNTFELNLSLNSADFDMSVFKHWGYVDGKNLIVNYNEKGFQINGSNLTGKLGVKVNF
QPDYLDIPITKKGQGAIIGLVILAVVFIFVFAIVVYIMRKNKKFKAAVNSYRARYMHFNGDLVTMAPSDMA
PALVDLLINENYVSSSAISATLFYLCHKGYYTLENPSHIKNEFSSKKNNQDLVFKRNYQMAPPETAHLKYF
MRWMSKYERNGVLALKTIRSKVQTRSGALDYKTSNAQFDQTVKREAKELGFYVNIENKTILSNEYYDEKLK
WKAYKKYLMSYVNRKNMEPIESIDEMLIYASVLIGSQNQLKKLLDSVNNSVYSSNSFNDNLFYYYYPFFMT
NYSLWGGINYEANKDYSSGDGGGFGGFSSGSDFGGGGGGGSGAF

>Mediterraneibacter glycyrrhizinilyticus
MANQDWERFGEEILRNVQEAIDSRDYSMLNRTISDSVNGAVDYFSHTVKNAGDAMNRNMRSRQQENQYNTG
GGYRYQGNPSDRFTGRSANRDGKYATGQHMNQSADRYANRNSDTNGAFQKYTANRTVGEKQTPVPTEYYRS
VTGNRAAGILLSVVGCLAAGVFLVTGLAMGISGLFMEETGFLILGAVLFCGIPAAAFGVLTGLGTKMLGRV
KRFRSYIRTLAGREFCNLEELEREVKKSRRFVVKDLEYLIEKGWFRQGHLDEQRTCLMISNQSYHQYTDLM
KRTKEQEEQRKREEAEAQRKRETSGSRNPQLTPEVREVIRSGEAYIQKIHACNEAIPGEEISAKISRMEML
VDKIFDRVEQEPESVEDLHRLMTYYLPTTVKLLEAYEDLDAQPIQGANILSSKQEIEKTLDTLNTAFEKLL
DSLFEEKAWDVSSDISVLNTMLAQEGLTEDDFK
```

>*Lachnospiraceae bacterium*
MANQDWERFGEEILRNVQKAIDSRDYSMLNRTISDSVNGAVDYFSHTVKNAGDAMNRNMRSRQQENQYNTG
GGYRYQGNPSDRFTGRSANRDGKQATGQYRDQSADRYANRNSDTNGAFQKYTANRTAGGKQTPVPTEYYRS
VTGNRAAGILLSVVGGLAAGVFLVTGLAMGISGLFMEETGFLILGAVLFCGIPAAVFGVLSGIGTKMLGRV
KRFRSYIRTLAGREFCNLEELEREVKKSRRFVVKDLEYLIEKGWFRQGHLDEQHTCLMISNQSYHQYTDLM
KRTKEQEEQRKREEAEAQRKRETSGSRNPQLTPEVREVIRSGEAYIQKIHACNEAIPGEEISAKISRMEML
VDKIFDRVEQEPESVEDLHRLMTYYLPTTVKLLEAYEDLDAQPIQGANILSSKQEIEKTLDTLNTAFEKLL
DSLFEEKAWDVSSDISVLNTMLAQEGLTEDDFK

>*Anaerocolumna sedimenticola*
MSRKNFSNLGDDIKDIVQDAVNTMNFQQLNRDIGNTVNGALDEVRGALGINRDRQNRDINGGRMDWDVTRG
NRRWNRSDQTYGQAQRQNNRTERNYNRQRSGQAPPQPVNNQYKMRQKNSNNGGSYPQVPVGRVSSILLTVF
GSIGFGGAGISAFVLALIGQLTGNLNFFGTIALSLLPILFISIFLMARGSRIRKRLKRFRRYLATFRNRSY
YSIKELSANTLLSQKFILKDLRKMISLGMFPEGRIDDQETCIMLNRESYEQYLELQKSIQMRNLEEQEKSK
NAVKQGSGQEMSRETSGSQGMNPELRKAIENGRNCIFKLREANEAIPGEEISRKLDRLEEVIGKIFNYVEL
HPEQLPEIEKFMEYYLPTTLKLVNAYREFDTQSVQGANITSAKSEIEATLDTINLAFEKLLDSLFEDAAMD
VSTDISVLETMLAQEGLTKEDFKVK

>*Rhodococcus kroppenstedtii*
MTRTRLVVAIVALGVVLGLTGCSTSSSADARFDGLVAGERVYDLTEFSLDGAETDALDRRLESLRAETGAD
VVVYVRELDADPDDTLDQVEALQQTWVAATGVDQDVAGAILVNREPGTTDEARAGIFVGSRFDDGNVPSDE
QEAIVEDALIPPLKDGDVAGSLNAGLDRLQSSIVNGPPVTALDRFADGPGSTWLPWTGLIVVLVGAVAAFS
LYRRRPRPSLPPDSPTTYRPDHHTDPAVATALVHRSNQASATSATVLALAADDAVAFEPDGKKHGKPTIRV
RLLDESKARGEVPRAVWGRLAEHAVDGVVDHAALTKVASNTRSIGRVVEAELDARGWRAHGTGPVRWAFGG
LALLGGAVMVGAAVVAAAGAPIMLVAAIPGGLLLVTGLIMTVAYPGLTAAGLDAARPWEAYRDGLKAAAKK
DDTAVDLDAALPDIVALGLGSAYRKRLESGPELRAFTAPAGLPLAVDPSVVNWAAFTATFGASSSSATVSG
GGAGGGGGAAGST

>*Candidatus Fischerbacteria*
MYHNYAKTILYFFLVVLFFSASSLFAVQVTLKWVEIDLALTKDGKADFVYKIRWTVSGSLHGFYFEGLSVQ
PYFDMETSYAFDDSNRKYPLSITEVSPGKYDVILANNKAISNGTATYIIHFAGDIARSGNLATTTSEFGEL
VVLNWAPPQWEYPMEHETVYVYYPIEIPGEKYTMDEATQLGFKTEKFMNDNYKLFYKGQLYQGIHYFTVGI
HKEGLSAYEKMQIQQYVPAQHFDISRLKPLVEEPPAYQPPPPATTTTATEKPAVTIYDFLYFIVMGGFYML
YGWRKGARQEKAIEEAPKIDWVSENWVPPKIQVASFRQKGKIAELDPAEALLLIGESPNTIFTKILMDLKE
KEAVYIRSYEPLNIIRKKPVSGLKYYEQGLMRCLLPDGTVDQNGLKALMTEIVNKVSEKCWDCDLEATKAY
YEKIIKNTPVEQLTPDQRRYYIYRDSSDYQRIHKQFPVLGAQYANYASFIKSDACHSACYVHTACHDACHS
ACHSACHSACHSACHSACHSACVSGGAH

>*Desulfovibrionales bacterium*
MVRNFAWLGILVWLLAGMALSVSAPAQARKGDVHFVETDVDLNADGSAVVAYTVQWRVVGDRLRGFVFSG
SDRLAVGRFSDRSHAVDDAGNRYGLEINRLQRDAYEILLERGQGVRSGHVTYQFWFPTDFAQAGYLEPTTA
EDGRELVVFNWSPVQWDEAERQRHYTLRVHTPLELPAHVLDARAYVLENDLVLTEPWVNERYRIDYQRGEH
DRLVLLFHRSAPGNRYHMRVQIYLPAVWFDVAALQVAPSGMVGQAVDSLRELTGRGRSEHGADSLFFGVNA
LFVPGAVVLLAFFYLLMVGKQRSMVKAHRGLDAIRWDTLDWTPPKLVLSNFRVKGKICKDLSPLEAAFYLE
IPFRQILSAMFTSLVREGYLEVTTEQPTLQAKVLRPPDAQLDIYERMFIHAFANDGQLSQQELEEIMNMAV
RAVQQKAWDCDVAATQAFWRERIAGYDSETRETAARENRWQDTEYNRWYYWYHNDHPRYQRRYACQEDPWR
HEAAIPVDSDHLQRSFINMSPSFDMNVCHDACHSACHSACHSACHSACHSACHSACVSGGSR

>*Streptococcus pyogenes*
MKKILMTLVLCFSLLGIRIKAADVDYSITNYEGQLLLSKENTARFEQKVTYQFDTSYNGQYISLGRAGHLP
AGFAIDQKPKVEVYQNGQQVPVSQEFSDLGDGYRLKLYNAGQAGDKVDVKVTWQLHHLLTAYQDVAELNLT
PISDWDKTLEKVSLTVTTPTDIQDSNLWAHRGYYQKKPQVLKEGNSRYQINAKNVSGQLELHAYWDKKALL
GKEPVDVSTSKKNKIVALETKISRRRALLQLLFGKVIPLVEVGFLLWQLIQFTRLKKQFNRYHLANHTDHS
YEVPEDLSPLVLTQAIYGQSFAYLSPTASESQKLLIPKGVTFEALVQATLLDLIDQKVLLLTKEEGKAYLE
ISQLDRVTDEEAAFLDMAFGNKVTLPVDQLFSQYHYDADTIKQLKKTYKGKKLEQEIRQSSEQVIKAMKKA
SAAITNNVLETIKKLNLPDTYRQVTPAEKRKSNSVQGLGCLLLILNSGLLIYLAIKESGLALIYLALMVLT
MCLGFYISLKLDQYKKLGIETPEGGVRLHQWQSFKNMIRDIDKFEDVAIEGLVVWNRVLVYATLFGYAKKV

ERYLKVHRIALPEVYQAVRPGELSMVMYATTPTFVSSLSSATTSSNFSVSSGGGISGGGGFSGGGGGGGGG
AF

>*Agrobacterium fabrum*
MKTIAARLLALFVFLGVAFPAFAEEFIRSYHSVVEVAADGKLTVTETITARAEGQNIKRGIFRDFPLYALD
ANNRRTRVDFNVVSVERDGAPENWRTENIDGGIRIYTGSAEHFLPTGEHIFQITYTTARQIRFFSDYDELT
WNVTGNGWQFPMGEISATITLPRGVKATDTAVFTGPLGAKGRDARILSEGNEVFFATTRPFSVGEGMTVAV
KLPKGAIAAPDTSQEAGWWLRDNLAILLSGGGLFVVLLYYLRAWFAVGRDPAKGVVVPRWDAPEGLSPALV
NYVDNRGFSGAGWTALSASALDLAVKGYVVLEDLKNSIVIRRTQKQAGAELPSGQKTLLSSIGSPGETLTI
DKAHGTEVEKVGKQFRAAIEKEHRGKYYRSNAGYIVFGILLSIAIIVATLVFGDLDENAIVAVMVFGFFGF
FFSVLSIGIGRQFSRGASLSKRIGGIVMLAFAGFVAFSVIGGIATQILLDVTHDTKSLALAAIGGIVLTNA
LFLFLMGAPTPLGRKLMDGIEGLRTYLTLAEKDRMNMAGAPAMSPQHFETLLPYAVALGVEKPWSRTFETW
LATAAAGAAVASYAPGWYAGSNYGSFGDRIGGFSTSMASTIASTIPQPVSSSGSSFSGGGGGGGSSGSGGGG
GGGGGW

>*Escherichia coli*
MMAGIYRCILLLIVGLFFSSLSYAKNTEIPSYEEGISLFDVEATLQPNGVLDIKENIHFQARNQQIKHGFY
RDLPRLWMQPDGDAALLNYHIVGVTRDGIPEPWHLDWHIGLMSIVVGDKQRFLPQGDYHYQIHYQVKNAFL
REGDSDLLIWNVTGNHWPFEIYKTLFSLKLPDIAGNPFSEIALFTGEEGDTYRNGRILEDGRIESRDPFYR
EDFTVLYRWPHALLGNAPAPQTTNIFSHLLLPSTSSLLICFPSLFLACGWLYLWKRRPQFTPVDVIETDVI
PPDYTPGMLRLDAKLVYDDKGFCADIVNLIVKGKIHLEDHYDKNQQILIRVNEGATRNNAVLLPAEQLLLE
ALFRKGDKVVLTGRRNRVLRKAFLRMQKFYLPRKKSSFYRPDAFLQWGGMAILAVILYGNLSPVGWEGMSL
VGDMFIMICWLLPFLFCSLELLFARDDDKPCVNRVIITLFLPLICSGVAFYSLYINVGDVFFYWYMPAGYF
SAVFLTGYLTGMGYIFLPKFTQTGQQRYAHGEAIVNYLARKEAATHSGRRRKGETRKLDYALLGWAISANL
GREWALRIAPSLSSAICAPEIARNGVLFSLQTHLSCGAYTSLLGRSYSGGGSGAGGGGGGGW

>*Bifidobacterium longum*
MEIGWNIPTTVKTNSIKFTVSFTMHDVATKWKDVATFQWEPFGENNQVPIGTVTGTVHFPKGVTASNSWTW
LHTEQTSETGRTKDGSFTFTVNDVKSGEYLDVVAAFDAAKAGDMARVETDDHLQELKEDEADDERFWRNLE
RQRATRRLNNWMTTISAGLIFGIIGVVAALRSSGRSRYRGSIEYWRDRPELSPASAAKLIDIVTSGDKPDI
SERQLVATLLSLAVKKVIAVFPGSADLYIGVDLKQVSPVELSQRIGSDPYKRNNARTNSTIVMLWQSDYGV
SEGFDESVFVESVFDGSGSGGSGSAASSVSDAHQPDLSASERALLYLLTVISERVGRTAFDMDDIKDACKD
WKDGYKELEKFTNACKTEFAAADIAPKTENGWLIPGAITVLIGGASIFINGLAGYLVAGALIGIPLMTVGI
FCFLMGNSYVLTDHGQQMTGQCLGLKRYMQGFSNFKYRGVADLTLWDWYMVYAAVFGISDRVMRELAMAYP
QVSDPEWLDANASDTVFYWNYRPYDWYGLRFYNGSAFADSVADSGLAGAVPAFGGTSFAAGFSDLGTQLSY
GFADITATINAASPSGGSGGFGGSSGGSGGGSFGGR

>*Prevotella ruminicola*
MKAKLTIVLLLLGVIQVLARPQLHDLDIRVVLDRNGDALITETRQMTIDSEGTECYIGLANMGPSIVKNLT
VTDETGARYVYTDWDVNQSRSWKQYKCGIVTTKRGFELCWGLGAEGERTYTTTYVMTGLVRGYPDACAIRH
VFLDTAVSPKPEHARVAITTADSTMVINEDSCGVWGFKFEGEIWMQDGFVMAETTKPMSSEAGLYLMVKFP
KSMFEPAVWENDTFENKKEEAFEGSDYVYDEDDEDDMSPFEWVLFILFYGGAALVVIGGAVWQMYRVWRAK
RQLNKGLMWYRGVPLNGDLQEANKILNAYKYFNSDYNNLMSACILKLISMGIITIETKPNSKGKMEPNFVM
HDYKDIDKQPVLMRQLYKMFKDAAGSDRILEPWELKSYMRSRTHQSAIDQFITTLHAKKDLSKYSPYDKDV
NEVFGLRKFLKEFTLLDERELKEVKLWKDYMIYATLFGIADKVIKEMKQVNPAYFDMDRVASTMADNMTLP
LIYSTLQRSTSSAVAAKAAREHRASGGGGHSSWGGGGGGFSGGGGGGGVR

>*Bacillus subtilis*
MKHSFFFFMFIAFFLVTGFSEAGKSFSIERADIRATVKENGDLSVEEVYTYDFKGSFEGATRSFSEETASR
LKNFKAYLLPDHRKKGDQATPLKTKKEDGTYYAYSASKDETKHVMFRYVIEDAAQKFEDTSILIHSFYQKA
NTDFGRVKIEVHLPDSVKARDIHAFLREKGNGKITKVSDSSVTYETGLYRAGTGSELRIYFPQSALKEAVR
HPSYQTKDELLKEERDEAKRYADRDERIAGADRMIWIFSGIVGFMVIMAVMLSFLKKRRRAMLSFAELESF
DPVLVAFLYKKGRFTDRDLLAGMLSLYQRGLVTMRKVQAEERFLDDPEAPDDTYQFEFSGSKADLPKPDQL
LIEKLFEQTGSDSYMFRLDSLSGPTEEECKHKEQLGKYERKRIILQNVVQKWTESVRQAPEFRELFQENRW
LRMLSLSLLSVHTGLLLFILYADVVHQAGFMAACVIFGFALSAGIVLAKSKLYTILFLAGLFLASLFTDSI
HTILYYGICVGLSILLIAFIPAVKESWYASICRRSILSWRNHLRKEDLFSGRSLSQNEKTMIYAIVLEVED
ESAGWFNQSANRLDYASPVLGMAAAGCLHYPFYSWQLPSSQEHAGAYDAGGPGAGGGDGAGAF

```
>Streptococcus agalactiae
MKKCFLAICLALSFFMVSVQADEVDYNIPHYEGNLTIHNDNSADFTEKVTYQFDSSYNGQYVTLGTAGKLP
DNFDINNKPQVEVSINGKVRKVSYQIEDLEDGYRLKVFNGGEAGDTVKVNVQWKLKNVLFMHKDVGELNWI
PISDWDKTLEKVDFWISTDKKVALSRLWGHLGYLKTPPKIRQNNNRYHLTAFNVNKRLEFHGYWDRSYFNL
PTNSKNNYKKKIEYQEKMIERHGFILSFLLRILLPSFFIIVTLFISIRVFLFRKKVNKYGQFPKEHHLYEA
PEDLSPLELTQSIYSMSFKNFQDEEKKTHLISQEQLIQSILLDLIDRKVLNYDDNLLSLANLDRASDAEID
FIEFAFADSTSLKPDQLFSNYQFSYKETLRELKKQHKASDLQTQMRRRGSNALSRITRLTRLISKDNINSL
RRKGISSPYRKMSSEESKELSRLKRFSYLSPLISFVVIIYTLFLNYFTYFCIYLLLFGVILLLNKIIFMMT
RKISNGYIVTEDGASRVYQWTSFRNMLRDIKSFDRSELESIVLWNRILVYATLFGYADRVEKALRVNQIDI
PERFANIDSHQFAISVNQSSNHFSTITEDVSHASNFSVNSGGSSGGFSGGGGGGGGGAF

>Lactobacillus casei
MKKKYLGLVALLSLLSVFAIWGQSVRADVDYDIDNVRAIARVNKDGSLTMHRTIKYSFDSDARGVYYKQNL
AVKQKLSNIQVKVDGQNIKAATTGKNNTYKLQKQGNSYNFKVYHRIKENDKVKVEYSYLIHRAITNYLDTA
ELNFKIVGNGWDTDLDHVRAEVIFPGAVKGLKAWAHGPLSGYTQVLPKEGKIIMTADDVAGDSGVEVHAIF
PTTVTSANQNIVKENKKRAIEKQEAALAKEANQKRQRKQMLSIGLMIISVLVGFVVVIRGFFIKKVGVKPK
IERDLVHNYEIPDISPTTAQILDEADKPNVKAFVAYLMQLAGKNKIKIEKYQTKHLKRTNYRITLVDDSVL
TDDLLDFIFNKVGDGKSFTTKDLRDYTSKKLGRRFDKWCDGQYKQVEDKDLLDKKYKKQRSNFRTGMLMGM
IASFAIWVISLMMANNIPSFVIIIGIMMIVLEVVAFIVGNSRLSIYTQKGALETDQVRGFKKMLNDIGQFK
MKDVGDLILWEDIMPYAVAFGLSKKVLKQLKIEFADELDAAPVLFYSGFYSSSSDSFEHSFERSFSSAVST
GSSSVSGSSGGFSGGSSGGFGGGSGGGAF

>Streptococcus gordonii
MYIEEASEGSASDDVEGIEWRIYVKKRLLFIISILFLVTLLIPFRGKAAEIDSRIEQYNGRLEVHQDNTAT
FIEEVTYVYDDPYNGQYITLGQAGKVPSNFEIESNPVVEIETNGKTKEPQSIEEVPIEDGKKLKIYNSGNS
GDQVKIKITWQLKNLLFLYPDVAELNWIPISDWEVGMDHVSFVVTSQPDSSARLVAHTGFFKKDPEVKRIE
NGFEVTLDSLGAKHHFELHGIWDRSLFSQSLTNDGGITNRRDSFEKQEQDIVRKTVFYQNLVYKILPVVFL
VIFVISIYYLIRYFKVTRQKTSFSDQARLYEVPQDLPPMLVALNIYDVDIEKVGPVQGKKGRLLFSNLIQA
TLLDLVDRGNLKYVTEGQSHRLEIVDYEGMAGFELTFVEMVFGDKSSVEPDTMFSAYQIDKKILKGVKDKD
DEAEVRKEGSDKRYRFIKDLRKLSNDIKEEEQRLGLHPHFRSLNKEEEKMRNRGCLLYLLVFLLLMFSLIG
FGFLFREFFWQYSLGFLLVLIIGIPLNAFVTKRSDNLLNEDYIDEVVEWRSFANMLRDIAKFDKTEVEGVI
LWNRLLVYATLFGYAKRVSKVMKVQDIHLENEELERFVLTDQSLHFAGGVDLLNSYVQTASSASTFSISSG
SDSGGFDGGGFSGGGGGGGGGGSF

>Streptococcus oralis
MKKTFLLLVLGLFCFLPLSVFAIDFKINSYQGDLYIHADNTAEFRQKIVYQFEEDFKGQIVGLGRAGKMPS
GFDIDPHPKVQAAKNGSELADVTSEVIEGADGYTVKVYNPGQEGDTVEVDLVWNLKNLLFLYDDIAELNWQ
PLTDSSGAIGKFEFHVRGDKGAEKLFFHTGKLFREGTIEKSSLDYTIRLDNLPAKRGVELHAYWPRTDFAN
ATDQGLKGNRLEEFNKIEDSIVKEKEQSKQLVTRVFPSILSISLLLSICFYFIYRRKTTPSVKYAKNHRLY
EPPMELEPMVLSEAVYSTSLEEVSPLTKGAGKFTFDQLIQATLLDVIDRGNVSIISEGDAVGLKLVKEDGL
SSFEKDCLNLAFSGKKEETLSNLFADYKVSDSLYRRAKVSDEKRIQAKGRQLKSSFEEVLKEMQEGVRNRV
TFWGLPDYYRPLTGGEKALQVGMGVLTILPLFIGFGLFLYSLDVYGYLYLLLPILGFLGLVLAVFYYWKLR
LDNRDGVLNEAGAEVYYLWTSFENMLREIARLDQAELESIVVWNRLLVYATLFGYADKVSHLMKVHHIQVE
NPDINLYVAYGWHSMFYHSSAQMSHYASVANTASTYSVSSGSGSSGGGFSGGGGGGSIGAF
```

Figure S7. Alignment of selected DUF2207 proteins

```
Candidatus_Fischerbacteria/1-525                  1 ----------------------MYHNYAKTIL-YFFLVVLFF-SA--SS----LFAVQVTLKWVEIDLALTKDGKADFVYKIRWTVSGS-L
Desulfovibrionales_bacterium/1-558                1 ----------------------MVRNFAWLGILVWLLAGMALSVS--AP----AQARKGDVHFVETDVDLNADGSAVVAYTVQWRVVGDRL
Mediterraneibacter_glycyrrhizinilyticus/1-45      1 ------------------------------------------------------------------------------------------M
Lachnospiraceae_bacterium/1-459                   1 ------------------------------------------------------------------------------------------M
Anaerocolumna_sedimenticola/1-451                 1 ------------------------------------------------------------------------------------------M
Bacillus_subtilis/1-631                           1 ----------------------MKHSFFFFMFIAFFLV------T-GFSE----AGKSFSIERADIRATVKENGDLSVEEVYTYDFKGS--
Prevotella_ruminicola/1-547                       1 ----------------------MKAKLTIVLLLLGVIQVL--A---RP-----------QLHDLDIRVVLDRNGDALITETRQMTIDSE--
Clostridium_N3/1-541                              1 ----------------------MKKVVAVLLFFSIFLSPF------IVKA----DSKSYTIENLVVNSTILRNGDVYVEEELTYYFNGD--
Lactobacillus_casei/1-597                         1 ----------------------MKKKYLGLVALLSLLSVF--AIWGQSVR----ADVDYDIDNVRAIARVNKDGSLTMHRTIKYSFDSD--
Streptococcus_oralis/1-629                        1 ----------------------MKKTFLLLVLGLFCFLP--------LSV----FAIDFKINSYQGDLYIHADNTAEFRQKIVYQFEED--
Streptococcus_gordonii/1-662                      1 MYIEEASEGSASDDVEGIEWRIYVKKRLLFIISILFLVTLL--IPF-RGKA----AEIDSRIEQYNGRLEVHQDNTATFIEEVTYVYDDP--
Streptococcus_mutans/1-633                        1 ----------------------MKKLLLFLATCLSLV-----TTS-FVSA----KDVDYNIRLYWGRLTLNKDNTATFQQDLVYDFASS--
Streptococcus_pyogenes/1-641                      1 ----------------------MKKILMTLVLCFSLL-----GIR--IKA----ADVDYSITNYEGQLLLSKENTARFEQKVTYQFDTS--
Streptococcus_agalactiae/1-627                    1 ----------------------MKKCFLAICLALSFF-----MVS--VQA----DEVDYNIPHYEGNLTIHNDNSADFTEKVTYQFDSS--
Bifidobacterium_longum/1-604                        -------------------------------------------------------------------------------------------
Rhodococcus_kroppenstedtii/1-510                  1 ----------------------MT--RTRLVVAIVALGVV-LGLTGCSTSSSADA-------RFDG--LVAGERVYDLT------------
Agrobacterium_fabrum/1-645                        1 ----------------------MKTIAARLLALFVFLGVA-F-----------PAFAEEFIRSYHSVVEVAADGKLTVTETITARAEGQNI
Escherichia_coli/1-630                            1 ---------------------MMAGIYRCIL--LLIVGLF-FSSLSYAKNTEIPSY-EEGISLFDVEATLQPNGVLDIKENIHFQARNQQI
```

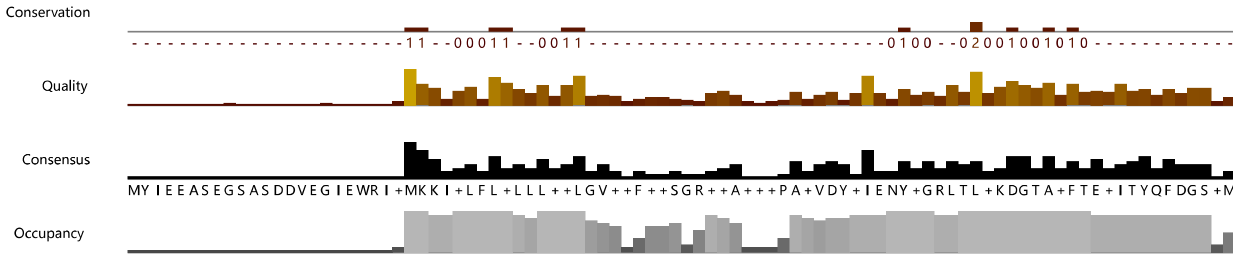


```
Candidatus_Fischerbacteria/1-525                101 YDVILANNK-AISN--------------GTATYIIHFAGDIARSGNLATTTSEFG-ELVVLNWAPP--QWEYPMEH--ETVYVYYPIEIPGE
Desulfovibrionales_bacterium/1-558              105 YEILLERGQ-GVRS--------------GHVTYQFWFPTDFAQAGYLEPTTAEDGRELVVFNWSPV--QWDEAERQRHYTLRVHTPLELPAH
Mediterraneibacter_glycyrrhizinilyticus/1-45     25 DYSMLNRTIS----------------------------------------------------------DSVNGAVDYFSHT--VKNAGDAM--
Lachnospiraceae_bacterium/1-459                  25 DYSMLNRTIS----------------------------------------------------------DSVNGAVDYFSHT--VKNAGDAM--
Anaerocolumna_sedimenticola/1-451                25 NFQQLNRDIG----------------------------------------------------------NTVNGALDEVRGA--LGINRDRQ--
Bacillus_subtilis/1-631                         101 TYYAYSA----SKDETK--------------HVMFRYVIEDAA---------QKFEDTSILIHSFY-QKANTDFGRVKIE--VHLPDSVK--
Prevotella_ruminicola/1-547                      92 SWKQYKC---GIVTTKRGFELCWGLGAEGERTYTTTYVMTGLV---------RGYPDACAIRHVFLDTAVSPKPEHARVA--ITTADSTM--
Clostridium_N3/1-541                            111 KIKIFSK----SSNERKT--------------FIFKYTIHGAV---------VKREDIGELYWAFYEVENNIPVNTFELN--LSLNS--A--
Lactobacillus_casei/1-597                       110 NFKVYHR---IKENDKVK--------------VEYSYLIHRAI---------TNYLDTAELNFKIVGNGWDTDLDHVRAE--VIFPG--A--
Streptococcus_oralis/1-629                      105 TVKVYNP---GQEGDTVE--------------VDLVWNLKNLL---------FLYDDIAELNWQPL-TDSSGAIGKFEFH--VRGDK-----
Streptococcus_gordonii/1-662                    133 KLKIYNS---GNSGDQVK--------------IKITWQLKNLL---------FLYPDVAELNWIPI-SDWEVGMDHVSFV--VTSQPD----
Streptococcus_mutans/1-633                      111 RAKVYNG---GHSGDRVV--------------LSLKWKLHHVT---------TIYSDIAELNWTPI-SDWDAPLDKVVLT--VKGPSN-S--
Streptococcus_pyogenes/1-641                    105 RLKLYNA---GQAGDKVD--------------VKVTWQLHHLL---------TAYQDVAELNLTPI-SDWDKTLEKVSLT--VTTPT--D--
Streptococcus_agalactiae/1-627                  105 RLKVFNG---GEAGDTVK--------------VNVQWKLKNVL---------FMHKDVGELNWIPI-SDWDKTLEKVDFW--ISTDK--K--
Bifidobacterium_longum/1-604                      1 ------MEIGWNIPTTV---------KTNSIKFTVSFTMHDVA---------TKWKDVATFQWEPFGENNQVPIGTVTGT--VHFPK--G--
Rhodococcus_kroppenstedtii/1-510                 71 DVVVYVRELDADPDDTL---------DQVEA---------------------------LQQTWVAATGVDQDVAGA------ILVNREPG--
Agrobacterium_fabrum/1-645                      105 R--IYT----GSAEHFL---------PTGEHIFQITYTTARQI---------RFFSDYDELTWNVTGNGWQFPMGEISAT--ITLPR--G--
Escherichia_coli/1-630                          114 S--IVV----GDKQRFL---------PQGDYHYQIHYQVKNAF---------LREGDSDLLIWNVTGNHWPFEIYKTLFS--LKLPDIAG--
```

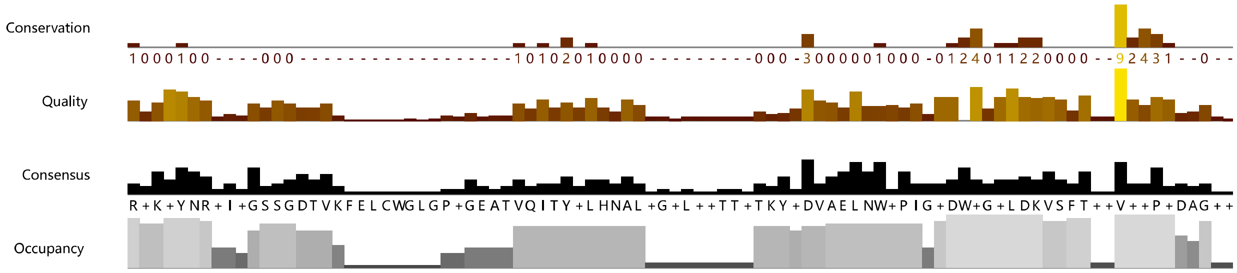


```
Candidatus_Fischerbacteria/1-525                223 KMQIQQYVP-AQHFDISRLKP---------------------LVEEPPAY--QPPPPATT--------TTATE--------------KPAV
Desulfovibrionales_bacterium/1-558              229 HMRVQIYLP-AVWFDVAALQV---------------------APSGMVGQAVDSLRELTG--------RGRSEHGADSLFFGVNALFVPGAV
Mediterraneibacter_glycyrrhizinilyticus/1-45    116 NGAFQKYTANRTVGEK--QTP-VPT---------------------EYYRSVTGNRAAGILLSVVGCLAAGVFLVTGLAMGISGLFMEETG
Lachnospiraceae_bacterium/1-459                 116 NGAFQKYTANRTAGGK--QTP-VPT---------------------EYYRSVTGNRAAGILLSVVGGLAAGVFLVTGLAMGISGLFMEETG
Anaerocolumna_sedimenticola/1-451                96 ------YNRQRSGQAP--PQP-VNNQYK---------M-RQKNSNNGGSYPQVPVGRVSSILLTVFGSIGFGGAGISAFVLALIGQLTGNLN
Bacillus_subtilis/1-631                         196 GSELRIYFP-QSALKEAVRHPSYQT--K-DELLKEERDEAKRYADRDER---IAGADRMIWIFS---------------------------
Prevotella_ruminicola/1-547                     205 GLYLMVKFP-KSMFEPAVWENDTFENKKEEAFEGSDYV----YDEDD-----EDDMSPFEWVLFIL-------------------FYGGAA
Clostridium_N3/1-541                            206 KLGVKVNFQ-PDYLDI---------------------P----ITKKG--QGAI-----IGLVIL---------------------------
Lactobacillus_casei/1-597                       206 GVEVHAIFP-TTVTSA--NQNIVKENKK-RAIEKQEAA----LAKEANQKRQRKQMLSIGLMII---------------------------
Streptococcus_oralis/1-629                      199 GVELHAYWP-RTDFANAT-DQGLKGNRL-EEFNKIEDS----IVKEKEQSKQL-----VTRVFP---------------------------
Streptococcus_gordonii/1-662                    228 HFELHGIWD-RSLFSQSLTNDGGITNRR-DSFEKQEQD----IVRKTVFYQNL-----VYKILP---------------------------
Streptococcus_mutans/1-633                      208 KLELHAYWK-RSDFAA--ATD-KSSKRL-SKFQALEAK----IARRQKFYPLL-----VGWLFP---------------------------
Streptococcus_pyogenes/1-641                    200 QLELHAYWD-KKALLGKEPVD-VSTSKK-NKIVALETK----ISRRRALLQLL-----FGKVIP---------------------------
Streptococcus_agalactiae/1-627                  199 RLEFHGYWD-RSYFNL--PTN-SKNNYK-KKIEYQEKM----IERHGFILSFL-----LRILLP---------------------------
Bifidobacterium_longum/1-604                    100 YLDVVAA---FDAAKAGDMARVETDDHL-QELKEDEAD-DERFWRNLER--QRATRRLNNWMTTIS---------------------AG
Rhodococcus_kroppenstedtii/1-510                143 QEAIVEDAL-IPPLKDGDVAGS-----L-------NAG-LDRLQSSIVN--GPPVTALD-RF-------------------ADGPGS
Agrobacterium_fabrum/1-645                      193 NEVFFAT---TRPFSVGE---G-------------MTV-AVKLPKGAIA--APDTSQEAGWW----------------------LRDNLA
Escherichia_coli/1-630                          204 RIE---S---RDPFY-RE---D-------------FTV-LYRWPHALLG--NAPAPQTTNIFSHLL------------------LPSTSS
```

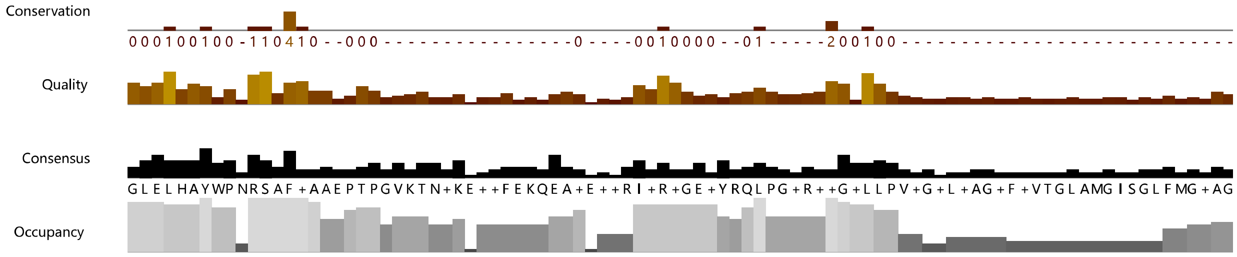


```
Candidatus_Fischerbacteria/1-525                315 IQVASFRQKGKIAE-LDPAEALLL-----IGESPNTIFTKILMDLKEKEAVYIRSYEP--LNIIR----KKPVSGLKYYEQGLM--------
Desulfovibrionales_bacterium/1-558              329 LVLSNFRVKGKICKDLSPLEAAFY-----LEIPFRQILSAMFTSLVREGYLEVTTEQPTLQAKVL----RPPDAQLDIYERMFI--------
```

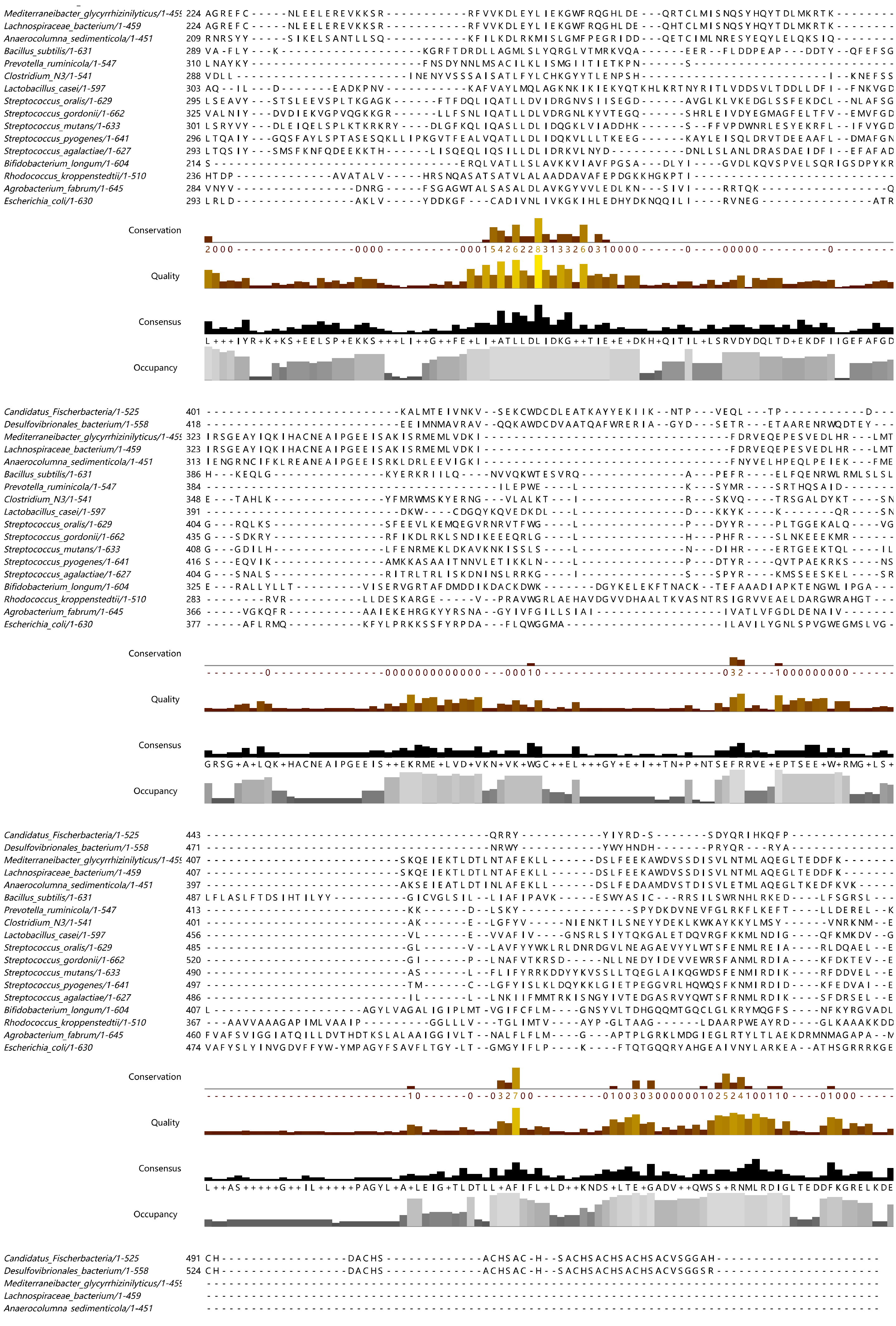
Mediterraneibacter_glycyrrhizinilyticus/1-459
Lachnospiraceae_bacterium/1-459
Anaerocolumna_sedimenticola/1-451
Bacillus_subtilis/1-631
Prevotella_ruminicola/1-547
Clostridium_N3/1-541
Lactobacillus_casei/1-597
Streptococcus_oralis/1-629
Streptococcus_gordonii/1-662
Streptococcus_mutans/1-633
Streptococcus_pyogenes/1-641
Streptococcus_agalactiae/1-627
Bifidobacterium_longum/1-604
Rhodococcus_kroppenstedtii/1-510
Agrobacterium_fabrum/1-645
Escherichia_coli/1-630
Candidatus_Fischerbacteria/1-525
Desulfovibrionales_bacterium/1-558
Conservation
Quality
Consensus
Occupancy

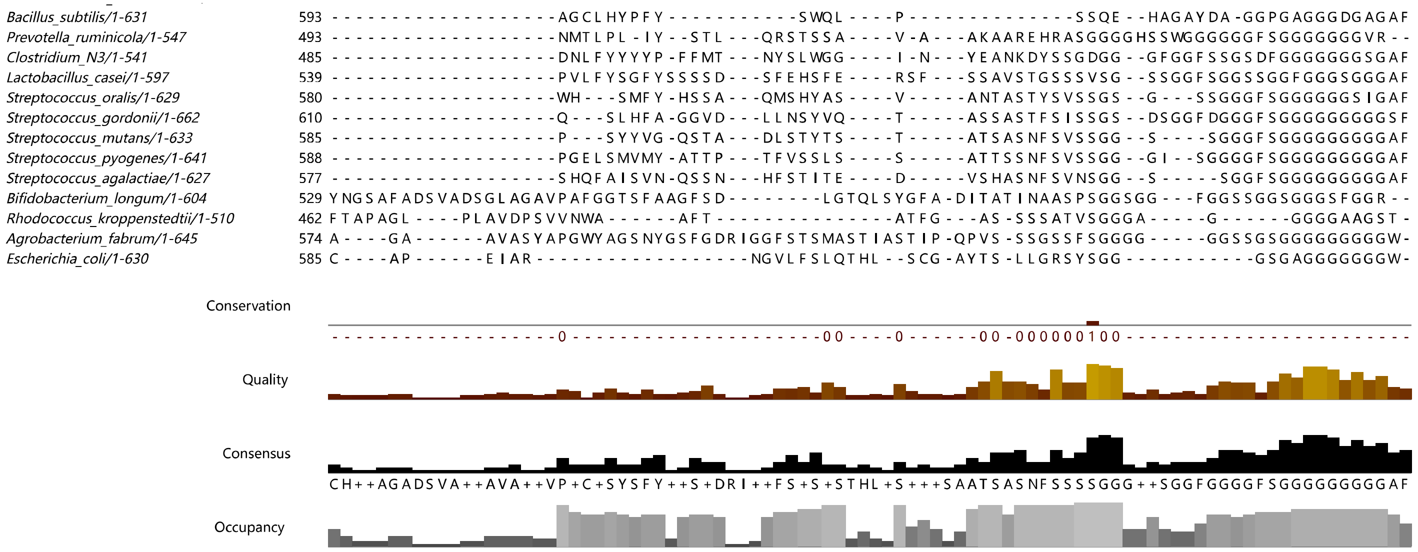

Bacillus_subtilis/1-631 593 -------------------AGCLHYPFY-----------SWQL----P--------------SSQE--HAGAYDA-GGPGAGGGDGAGAF
Prevotella_ruminicola/1-547 493 -------------------NMTLPL-IY--STL---QRSTSSA----V-A---AKAAREHRASGGGGHSSWGGGGGGFSGGGGGGGVR--
Clostridium_N3/1-541 485 -------------------DNLFYYYYP-FFMT---NYSLWGG----I-N---YEANKDYSSGDGG--GFGGFSSGSDFGGGGGGGSGAF
Lactobacillus_casei/1-597 539 -------------------PVLFYSGFYSSSSD---SFEHSFE----RSF---SSAVSTGSSSVSG--SSGGFSGGSSGGFGGGSGGGAF
Streptococcus_oralis/1-629 580 -------------------WH---SMFY-HSSA---QMSHYAS----V-----ANTASTYSVSSGS--G---SSGGGFSGGGGGGSIGAF
Streptococcus_gordonii/1-662 610 -------------------Q---SLHFA-GGVD---LLNSYVQ----T-----ASSASTFSISSGS--DSGGFDGGGFSGGGGGGGGGSF
Streptococcus_mutans/1-633 585 -------------------P---SYYVG-QSTA---DLSTYTS----T-----ATSASNFSVSSGG--S----SGGGFSGGGGGGGGGAF
Streptococcus_pyogenes/1-641 588 -------------------PGELSMVMY-ATTP---TFVSSLS----S-----ATTSSNFSVSSGG--GI--SGGGGFSGGGGGGGGGAF
Streptococcus_agalactiae/1-627 577 -------------------SHQFAISVN-QSSN---HFSTITE----D-----VSHASNFSVNSGG--S-----SGGFSGGGGGGGGGAF
Bifidobacterium_longum/1-604 529 YNGSAFADSVADSGLAGAVPAFGGTSFAAGFSD--------LGTQLSYGFA-DITATINAASPSGGSGG---FGGSSGGSGGGSFGGR--
Rhodococcus_kroppenstedtii/1-510 462 FTAPAGL----PLAVDPSVVNWA------AFT---------------ATFG---AS-SSSATVSGGGA-----G------GGGGAAGST-
Agrobacterium_fabrum/1-645 574 A----GA------AVASYAPGWYAGSNYGSFGDRIGGFSTSMASTIASTIP-QPVS-SSGSSFSGGGG-----GGSSGSGGGGGGGGGW-
Escherichia_coli/1-630 585 C----AP------EIAR-----------------NGVLFSLQTHL--SCG-AYTS-LLGRSYSGG-----------GSGAGGGGGGGW-
Conservation
-------------------0--------------------00----0------00-000000100---------------------------
Quality
Consensus
CH++AGADSVA++AVA++VP+C+SYSFY++S+DRI++FS+S+STHL+S+++SAATSASNFSSSSGGG++SGGFGGGGFSGGGGGGGGGAF
Occupancy
